# Acromioclavicular dislocation associated with fracture of the coracoid process: a series of cases and review of the literature

**DOI:** 10.1007/s00264-025-06435-1

**Published:** 2025-02-24

**Authors:** Andrés Combalia, Maite Combalia, Ernest Muñoz-Mahamud

**Affiliations:** 1 Departament de Cirurgia i Especialitats Medicoquirúrgiques, Facultat de Medicina i Ciències de la Salut, Universitat de Barcelona (UB), Barcelona, Spain; 2 Department Orthopedic Surgery and Trauma, Hospital Clinic of Barcelona, University of Barcelona and Hospital Quirón Barcelona , Barcelona, Spain; 3 Institut d’Investigació Biomèdica August Pi i Sunyer (IDIBAPS), Barcelona, Spain; 4 Orthopedic Surgery and Trauma, Pius Hospital, Valls, Tarragona Germany

**Keywords:** Acromioclavicular dislocation, Coracoid process fracture

## Abstract

**Purpose:**

Complete acromioclavicular (AC) dislocation associated with fracture of the coracoid process (CP) is uncommon. The strong coracoclavicular ligaments, instead of rupture, may avulse the CP near its base, and with disruption of the AC joint may allow complete dislocation of the clavicle. We report ten cases, one of the largest series in literature, and reviewed the findings and treatment previous reported cases, to allow potential readers to establish the most appropriate treatment.

**Methods:**

We have prospectively collected those cases in which we had identified an association of an AC dislocation with a fracture of the CP, as well as retrospectively reviewed the records that were coded as AC dislocations and CP fracture looking for this association in the senior author institutions. A literature search was completed on PubMed, Web of Science and Scholar Google, using a sensitive search strategy.

**Results:**

We have collected a total of ten patients with the association of a CP fracture to an AC dislocation in a period of twenty-five years. A review of the cases reported in literature shows a great variability in treatment methods from conservative to more surgically in recent years.

**Conclusions:**

When an AC dislocation is identified by clinical examination and X-rays, one should be aware of a possible fracture of the CP. It is possible this association to be more frequent than shown in literature because of the CP fracture can easily be missed out or mistaken with an unfussed epiphysis in routine anteroposterior radiography. Multiple approaches have been opted for by surgeons to deal with this combined injury and are the basis of this review.

## Introduction

Acromioclavicular (AC) dislocation is a common injury. In 1946, Urist reviewed 41 complete AC dislocations and reported two cases of associated fractures of the coracoid [1]. A concomitant fracture of the coracoid process (CP) seems to be an unusual association, with few reported in the English literature. In 1995 with the presentation of a case of a 12-year-old boy we reviewed the literature and founded only 29 cases reported [2–16]. Since then a few cases have been reported as a case report [17–54] or short series [55–56,57]. As a result of the rarity of these combined injuries there are no clear recommendations on the most appropriate treatment and the choice between surgical and non-surgical treatment remains a matter of debate [58].

In routine radiography the fracture of the CP might be unnoticed or be mistaken for an unfused epiphysis [59]. The purpose of this article is to report nine new cases and to review the findings and the treatment carried out in published cases.

## Materials and methods

We have prospectively collected those cases in which we had identified an association of an AC dislocation with a fracture of the CP, as well as retrospectively reviewed the records that were coded as AC dislocations and CP fracture looking for this association in the senior author institution. A literature search was completed on PubMed, Web of Science and Scholar Google, using a sensitive search strategy. Studies in Chinese (*n* = 2) or German (*n* = 1) were excluded from the review. This research has been approved by the institutional research ethics committee of the senior author, in line with the principles of the Declaration of Helsinki.

## Results

### Demographics data

We have collected a total of ten patients with the association of a CP fracture to an AC dislocation in a period of twenty-five years and their demographic data are shown in Table 1. The first case of this series has been published in 1995 [2]. Since then, we have been able to diagnose and treat nine new cases -ten in all-, nine males and one female, with a mean age of 28.8-year-old (range 12–54).

**Table 1:**
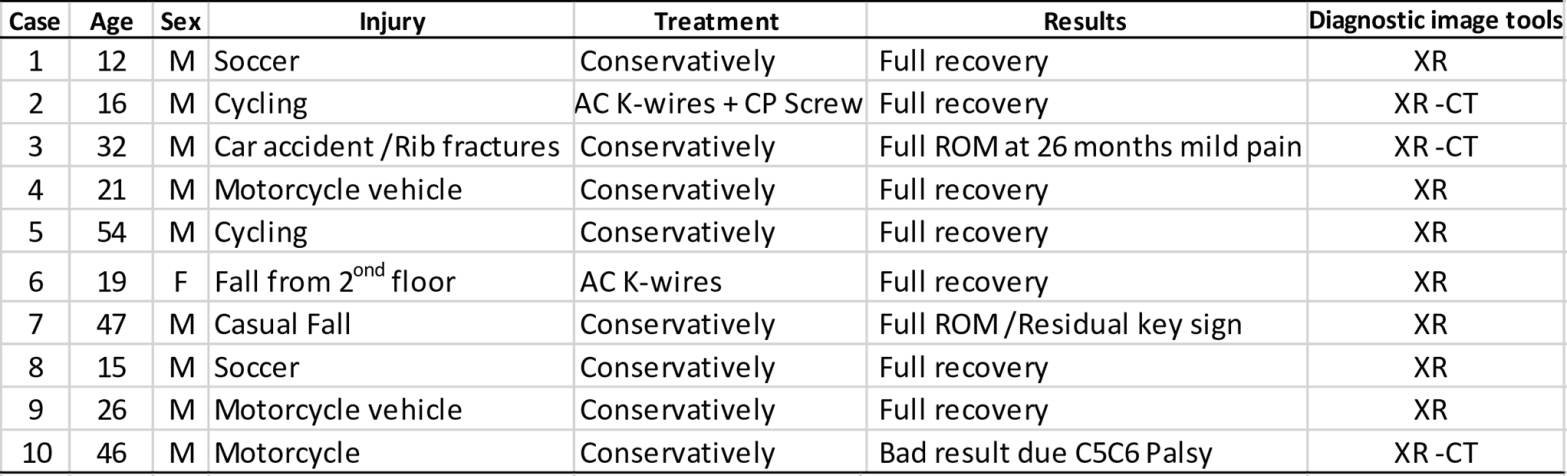
Demographic data of ten patients with acromioclavicular dislocation associated to a Coracoid process fracture. (M: male; F: female; AC: acromioclavicular; CP: coracoid process; ROM: range of motion; XR: Plan radiographs; CT: computed tomography)

### Injury causes

Four injuries were due to motor vehicle accidents -one patient had chest trauma, a second patient had a C5-C6 brachial plexus palsy-, four due to sport lesions, one patient precipitates from a second floor and another injured by casual fall. Eight patients, including the patient with chest trauma, were treated conservatively.

### Diagnostic image tools

The diagnosis of the injury was made by X-rays and in three patients it was confirmed by CT.

### Treatment approaches

**Fig. 1:**
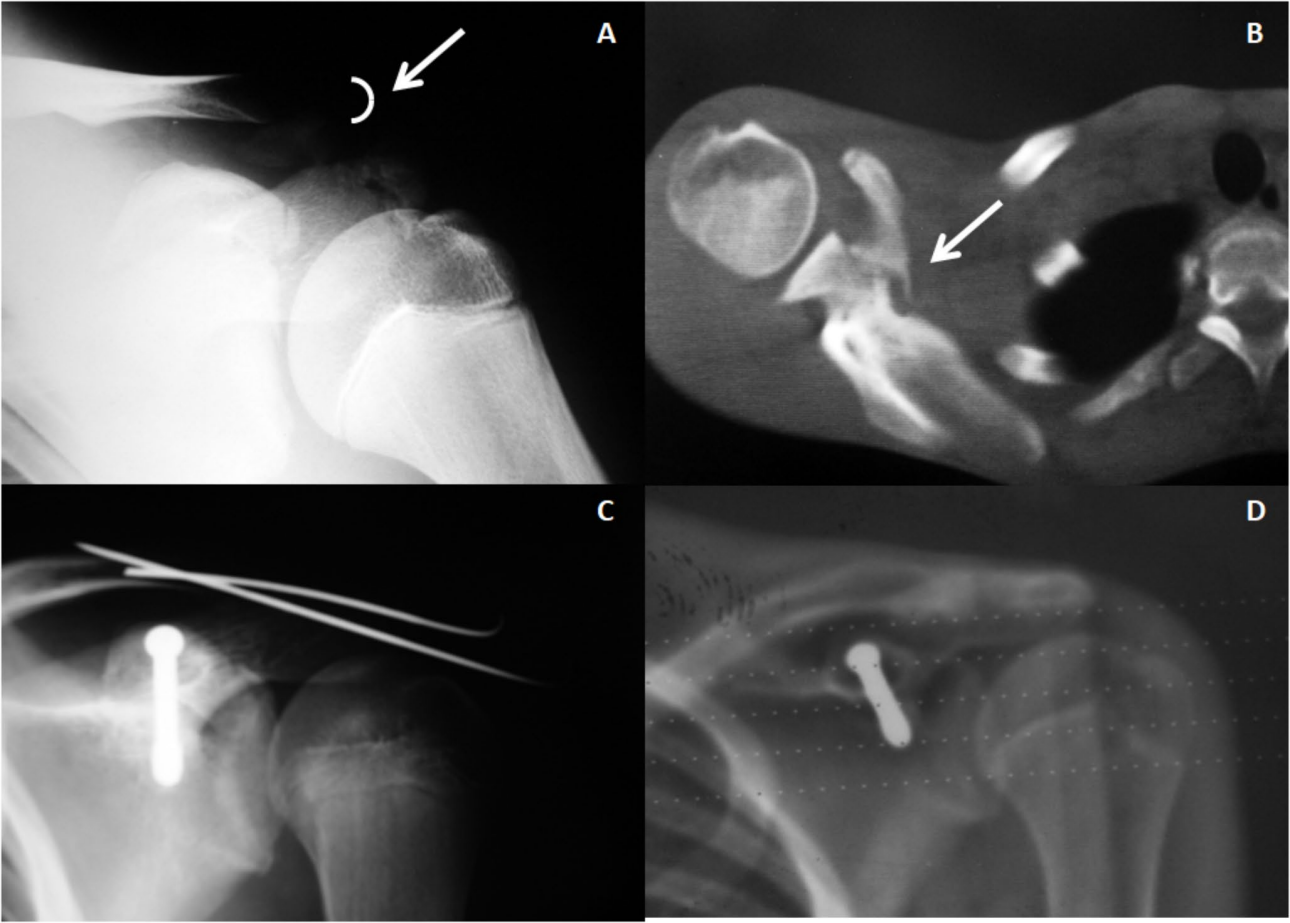
Case 2 Table 1. A 16 years-old boy: 1**A**: Antero-posterior radiograph showing an AC separation (white arrow), there is no clear vision of the CP; 1**B**: A CT scan shows a type I base associated CP fracture; (white arrow indicating the limits of the distal end of the clavicle drawn in white); 1**C**: Surgical treatment was performed with a screw fixation of the CP fracture and AC temporary fixation with Kirschnner wires; 1**D**: Radiological appearance at three months follow-up

**Fig. 2:**
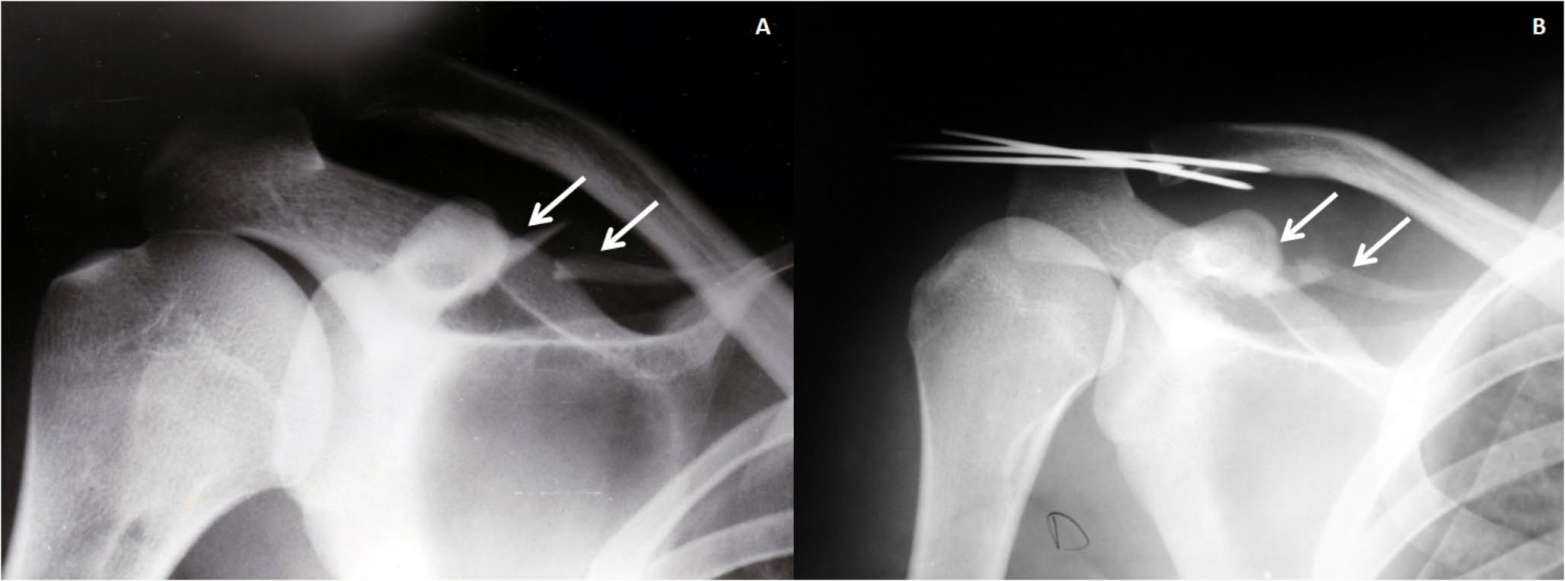
Case 6 Table 1. A 19 years-old girl: 2**A**: Antero-posterior radiograph showing an AC separation with and CP base and superior border of the scapula fracture; 2**B**: Treatment was made with an AC fixation with Kirschnner wires without surgical fixation of the CP

Two patients were treated surgically, a 16 years-old boy treated by means of a coracoid screw fixation and Kirschnner wires at AC joint (Fig. 1) and a 19 years-old girl in which a Kirschnner wires fixation at AC joint was performed (Fig. 2) (Table 1, cases 2 and 6). Eight patients (Mean age 31.6 y, range 12–54), included the patient with chest trauma (Table 1, case 3), were treated conservatively by means of a sling.

**Fig. 3:**
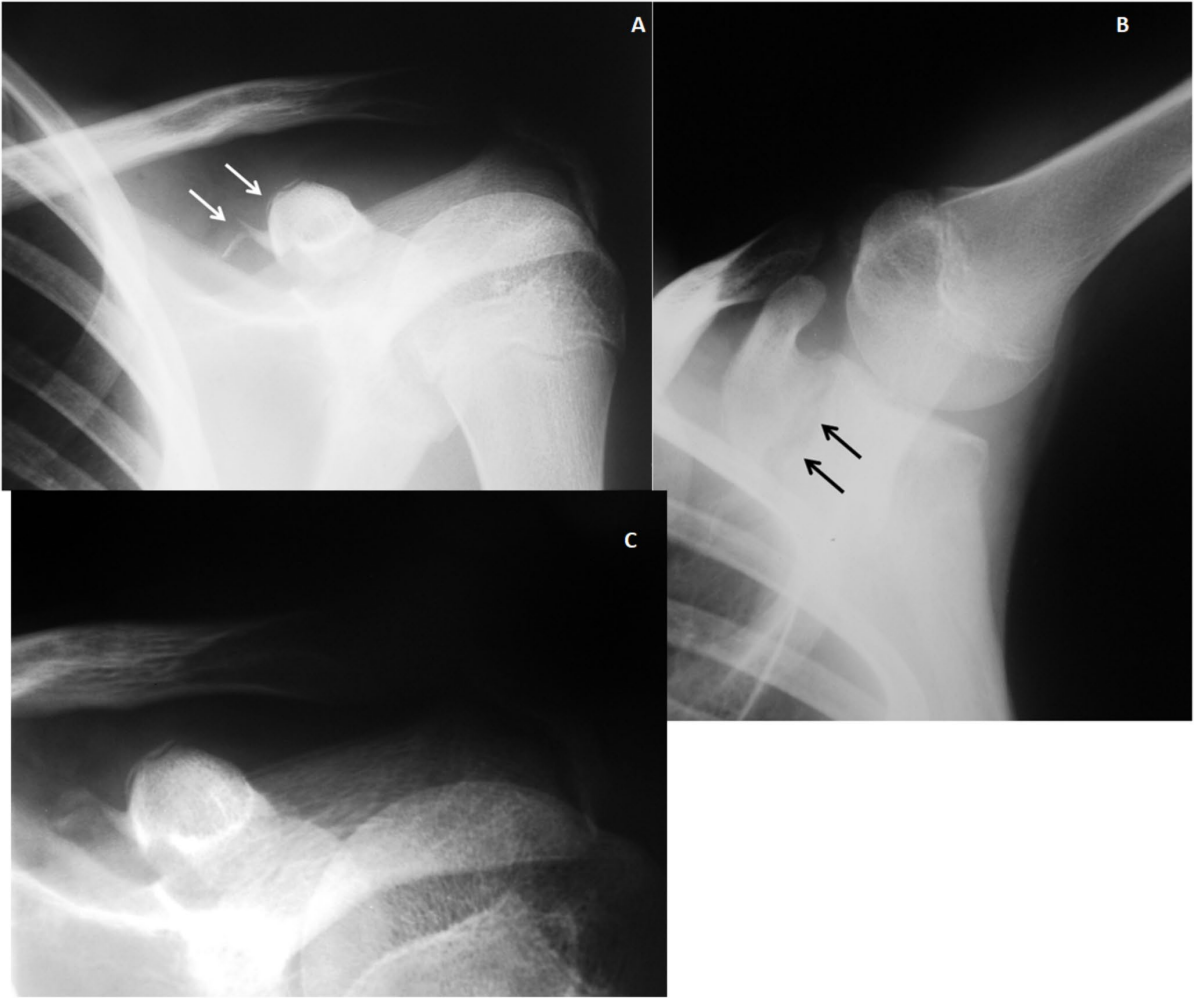
Case 8 Table 1. A 15 years-old boy: 3**A**: Antero-posterior radiograph showing an AC dislocation associated with a fracture of the superior border of the scapula that suggested a CP base fracture (white arrows); 3**B**: An abduction view radiograph reveals a fractured CP (black arrows); 3**C**: Conservative treatment by mean of Robert-Jones bandage was performed with good clinical result

Both the conservative and surgical treatment provided good results to the patients (Fig. 3 Case 8), not being able to establish that there was any difference except for the patient with brachial plexus injury whose shoulder function was compromised (Table 1 case 10). Two patients treated conservatively have residual pain or subtle AC minor piano key sign.

## Discussion

Isolated AC dislocations account for 9 to 12% of shoulder dislocations [59] and CP fracture accounts for 0-8% of scapular fractures reported in studies using plain radiography [60]. Coracoid Fracture associated with AC separation is uncommon. Urist called attention to these associated injuries in 1946 in a review of the subject of AC dislocations and reported two cases [1]. In another series of 116 AC dislocations, there was no association with fracture of the CP [61]. In 1995, with the presentation of a case of a 12-year-old boy, we reviewed the literature and found only 29 cases reported [2]. Since then, we have been able to diagnose and treat nine new cases -ten in all-. We did a thorough review of literature in 1995 and again today until 2024 for the purpose of this review. We have been able to find a total of 108 reported cases summarized in Table 2 (not including those in Chinese or German language).

The clinical results obtained in the patients of the reported series coincide with the results of the literature review. The surgical treatment carried out in two patients, either with fixation of both lesions or only with fixation of the acromioclavicular joint, yielded good results in the medium and long term (Figs. 1 and 2). Conservative treatment yielded good results in patients below eighteen years old. In adult patients treated conservatively (mean age 37.7 y- range 21 to 54 years) a satisfactory result was obtained in four of the six patients. In one of them a slight acromioclavicular subluxation persisted and the result of the patient in whom a C5-C6 paralysis was associated was poor without relation to the injury itself. These results are comparable to those reported by various authors who report isolated cases. Conservative treatment and surgical treatment seem to provide similar results. Broekman et al. in a recent systematic review (2023) of 37 patients previously published, communicates that surgical treatment was provided to 22 out of 37 patients (59.5%), and 15 patients had nonsurgical treatment (40.5%). The conclusion of this systematic review was that existing literature does not indicate that one treatment option is superior, and more data are needed to guide evidence-based decisions on this rare injury [57].

### Demographics data and Injury causes

Injury usually occurs in patients in the third and fourth decade of life (mean age of 103 injured whose age was recorded was 29.8 years -range 9 to 59-). Of these cases, 83 (80.6% -83 from 103 patients-) were male, 20 (19.4%) were female, and gender was not reported in the remaining five patients.

Mechanisms of injury could be seen in Table 2, traffic accidents, fall from height or sport injuries being the most frequent causes. These findings are consistent with the epidemiologic characteristics of the isolated AC dislocation in previous studies. The mechanism of injury is comparable to that of AC joint dislocations, except that instead of a rupture of the coracoclavicular (CC) ligaments, the tensile forces transmitted by these ligaments cause a fracture in the CP, allowing the vertical displacement of the clavicle [4, 8, 9, 50, 62]. Some authors believe that it is similar to AC dislocation, and thus defined it as variant AC dislocation [62].

### The Superior shoulder suspensory complex (SSSC)

**Fig. 4:**
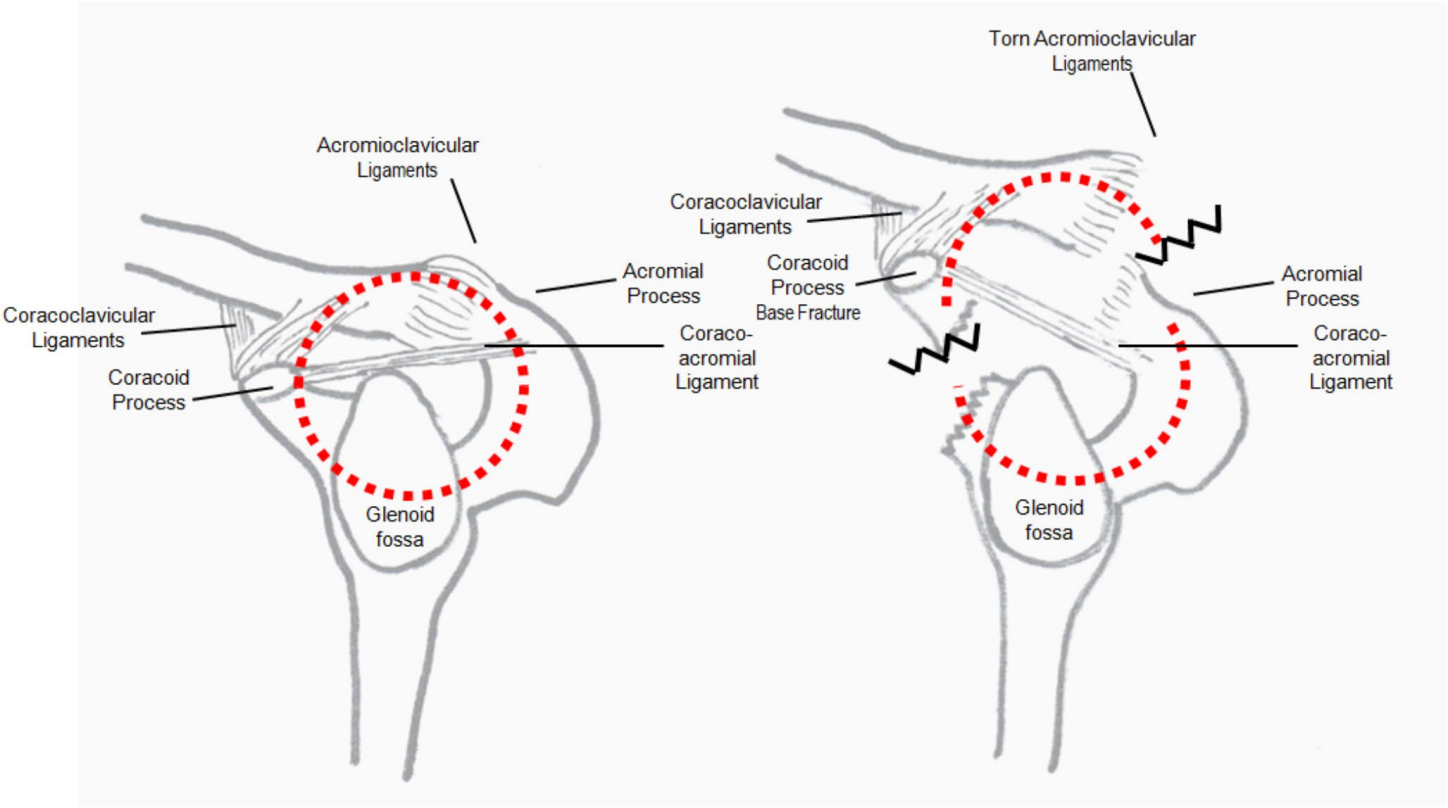
Schematic representation of the superior shoulder suspensory complex (SSSC) showing a lateral view of the bony soft-tissue ring (left) and rupture of the SSSC in two points: AC dislocation and base of the coracoid fracture (right)

The CP plays an important role in AC joint stability through the CC ligaments and is also the site of origin for the conjoined tendon of the coracobrachialis and short head of the biceps as well as the insertion of the pectoralis minor and coracoacromial ligaments. The AC joint and its stabilizing ligaments are part of the superior shoulder suspensor complex (SSSC), a bone and ligament ring structure, described by Goss in 1993 [63] whose integrity is essential to maintain the stability and biomechanics of the shoulder. The SSSC is a bony and soft tissue ring consisting of the acromion process, AC joint, distal clavicle, CC ligaments, CP, and glenoid (Fig. 4). These structures play an important biomechanical role in maintaining a stable upper extremity– axial skeletal connection [64]. According to this model, isolated SSSC lesions are relatively frequent and do not significantly alter the anatomic and functional stability of the ring. However, lesions that affect 2 or more points are much rarer and compromise the integrity of the ring, causing a potentially unstable situation that can lead to complications such as delayed union, loss of strength, and even degenerative arthritis [50, 63, 65]. Therefore, although it is a controversial issue, some authors advocate for surgical treatment of these combined lesions, whereas others prefer conservative treatment. Besides, in cases where surgery is chosen, the suitability of fixing one or both injured points is also controversial [55–57]. In isolation, each fracture is generally minimally displaced and can be managed non-operatively. In combination, however, each disruption can make the other unstable,

### Diagnostic image tools

**Fig. 5:**
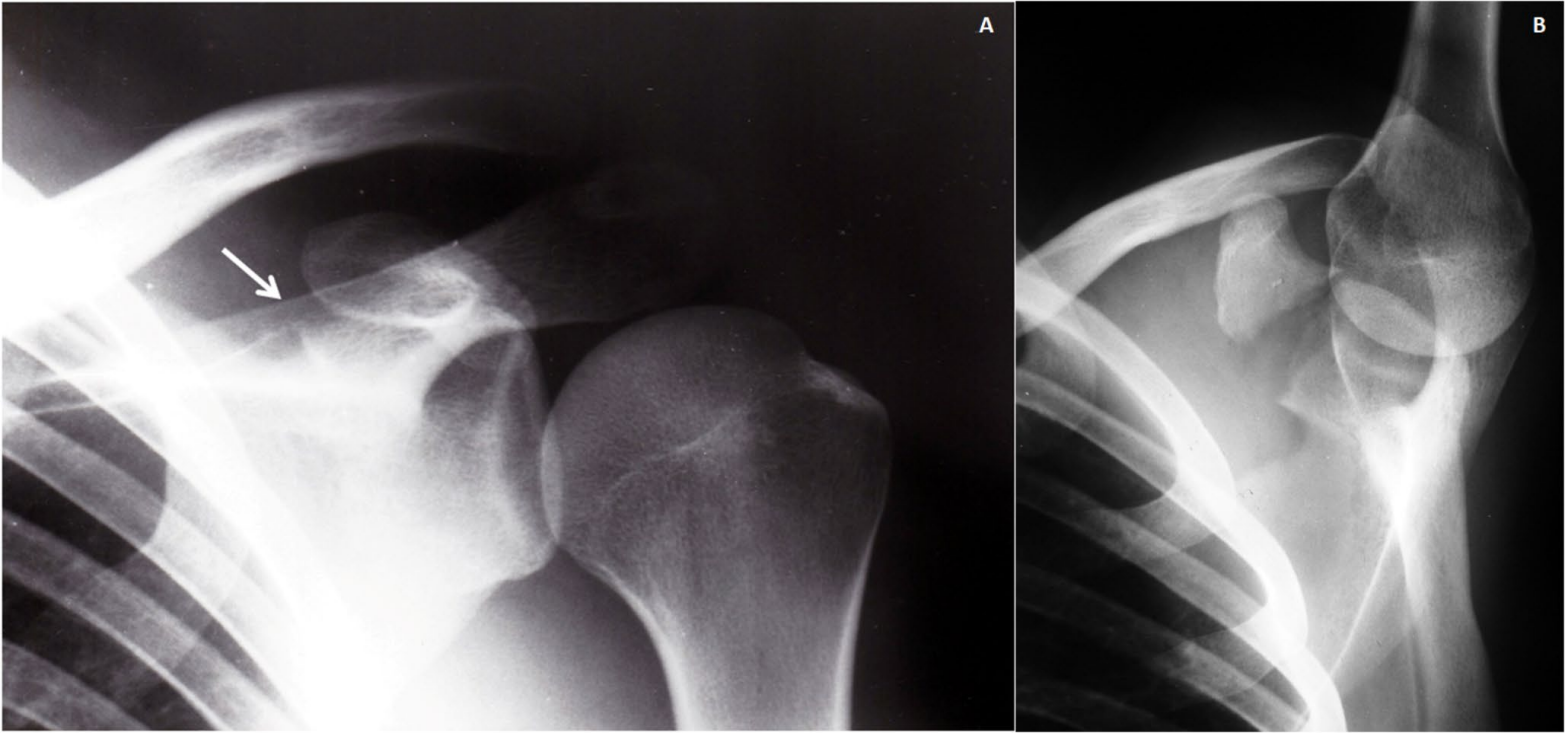
Case 3 Table 1. 4**A**: Antero-posterior radiograph of the left shoulder of a 32 years-old male shows an acromioclavicular dislocation with and associated CP base fracture (arrow). 4**B**: An abduction view shows more clearly the displaced CP base fracture without overlapping with other bony structures. Coracoclavicular distance is normal confirming CC ligaments were intact

The diagnosis of the coracoid fracture may be missed because the CP normally projects upward and medially, and then forward and laterally appearing foreshortened in the standard frontal view. In the same way, a fractured CP is easily overlooked when attention is directed toward the more obvious AC separation [12, 32]. High clinical suspicion is required. When an AC dislocation is observed in simple radiography, it is important to determine if the CC space is altered, and if it is maintained, we should seek an associated coracoid fracture. The presence of an AC dislocation could initially hide a CP fracture on regular AP and lateral radiographs. However, specific projections including Zanca, Stryker notch, axillary and 30° cephalad view should be considered to evaluate the CP status [4, 8, 9, 59, 66]. Axillary films may reveal the fracture, but it is a painful procedure in acute cases [10, 11, 67]. Taga et al. recommended an abduction view that clearly scans the CP without overlapping with other bony structures (Fig. 5) [12]. Even so, performing a computed tomography (CT) scan may be necessary for a definitive diagnosis and assessment of associated lesions [50]. In recent years and publications, CT has demonstrated its usefulness in the best diagnosis of these combined lesions [55, 56, 62]. In the present series, CT was only used in three patients, while in the other seven, the diagnosis could be made with a simple X-ray.

A CT scan provides a clear picture of both the CP fracture and the AC joint dislocation. One may have an idea about the CC ligament injury by measuring the distance between CP and AC joint in plain radiography. However, Magnetic Resonance Imaging (MRI) is the best way to assess the continuity of CC ligaments. These combined injuries pose many challenges to the trauma surgeon. Orthopaedic surgeons often fail to diagnose CP fracture in plain radiographs when an obvious AC dislocation is associated. In the last decades, the number of articles reporting many cases of CP fracture has been increasing due to increased awareness of coracoid fracture and advances in imaging techniques and methods such as CT and MRI [60].

In AC dislocations with CP fractures with intact CC ligaments, CP fractures are most frequently associated with type III or V AC dislocations [53, 55]. The importance of recognizing a coracoid fracture in AC is not only necessary to achieve a correct diagnosis but also is related to the treatment. One surgical method of repairing isolated AC separation consists in fixation of the clavicle to the coracoid with a lag screw or suture. This naturally would be contraindicated if the CP is not intact [68].

### The coracoid fracture and coracoclavicular ligaments

According to Ogawa et al., CP fractures can be classified into 2 types with respect to their location in relation to the CC ligaments, with type I fractures being proximal to the insertion of CC ligaments and type II fractures, distal [69]. Type I, located proximal to the CC ligament attachment, disrupt the scapuloclavicular connection and would be more unstable and often require an open reduction and internal fixation; type II could be caused by contractions of the coracobrachialis muscle, the short head of the biceps, or the pectoralis minor and is usually managed conservatively. On the other hand, AC dislocations can be classified into 6 types following Rockwood classification [59]: type I and II are frequently treated conservatively; type III can be debatable; and type IV, V, and VI require surgical management [70]. The association of AC dislocation and CP fracture simulates a complete disruption of the AC and CC ligaments which would bear resemblance to a type III or V AC injury or more. In fact, the most common association is a Type I CP fracture associated with a type III AC dislocation, occurring in up to 60% of the cases [40, 49, 53, 55].

The rupture of CC ligaments associated with AC dislocation and CP fracture is an exceptional entity that was observed in nine of the 108 reported cases in literature (8.33%), two under fifteen years-old (Table 2). The CC ligaments are usually intact when there is a CP fracture. However, some authors reported a concomitant simultaneous injury of the CC ligaments. In 1976, Zetttas and Muchnic [7] described a case in a 13,5 years-old boy in which the lesion was not suspected prior the operation and where it was found a CP fracture and a disruption of CC ligaments, repairing both lesions. Wilson and Colwill [14] reported the first case of this unusual triple lesion in a 20-year-old adult man, being the second adult case of 28 years-old reported by Wang et al. in 1994 [21, 71, 72]. Posteriorly we could find a description of these exceptional simultaneous triple lesion by six more authors [27, 28, 36, 43, 47, 51]. The treatment of this triple association injury is not yet unequivocal even if most authors prefer the surgical means [47, 51].

### Treatment approaches

Published cases include both surgical and conservative treatment for this double combined injury. According to the current literature, in these concomitant injuries, it seems there are no differences in the long-term outcomes of patients treated conservatively or surgically (Table 2). However, some earlier reports mentioned that AC joint pain and cosmetic symptoms persist after conservative treatment [9, 50]. The publications of recent years favour more the surgical treatment of the lesion [50, 55, 56, 62]. It seems logical to consider surgical treatment in those patients with significant displacement considering the potential instability of the combination of injuries.

Several surgical methods have been reported in the literature. Some authors choose to only address the AC joint [5, 10, 11, 13, 22, 40, 41, 49, 50, 53, 55], while others have found that AC can be reduced indirectly after CP reduction by surgery [7, 19, 30, 47]. In some cases, or series, authors prefer to fix both injuries, performing a fixation of the coracoid fracture and the AC dislocation to achieve both horizontal and vertical stability [7, 14, 27–29, 33, 42, 53, 56, 62]. Among the authors who opt for the surgical fixation of a single point of the ring, the procedures described consist in fixation of the CP with a screw whereas others perform fixation of the AC dislocation using Kirschner wires, a clavicular hook plate or through a Dewar-Barrington procedure, only described by Ishizuki et al. [10] and Samba et al. [51] (Table 2 and 3).

**Table 2:**
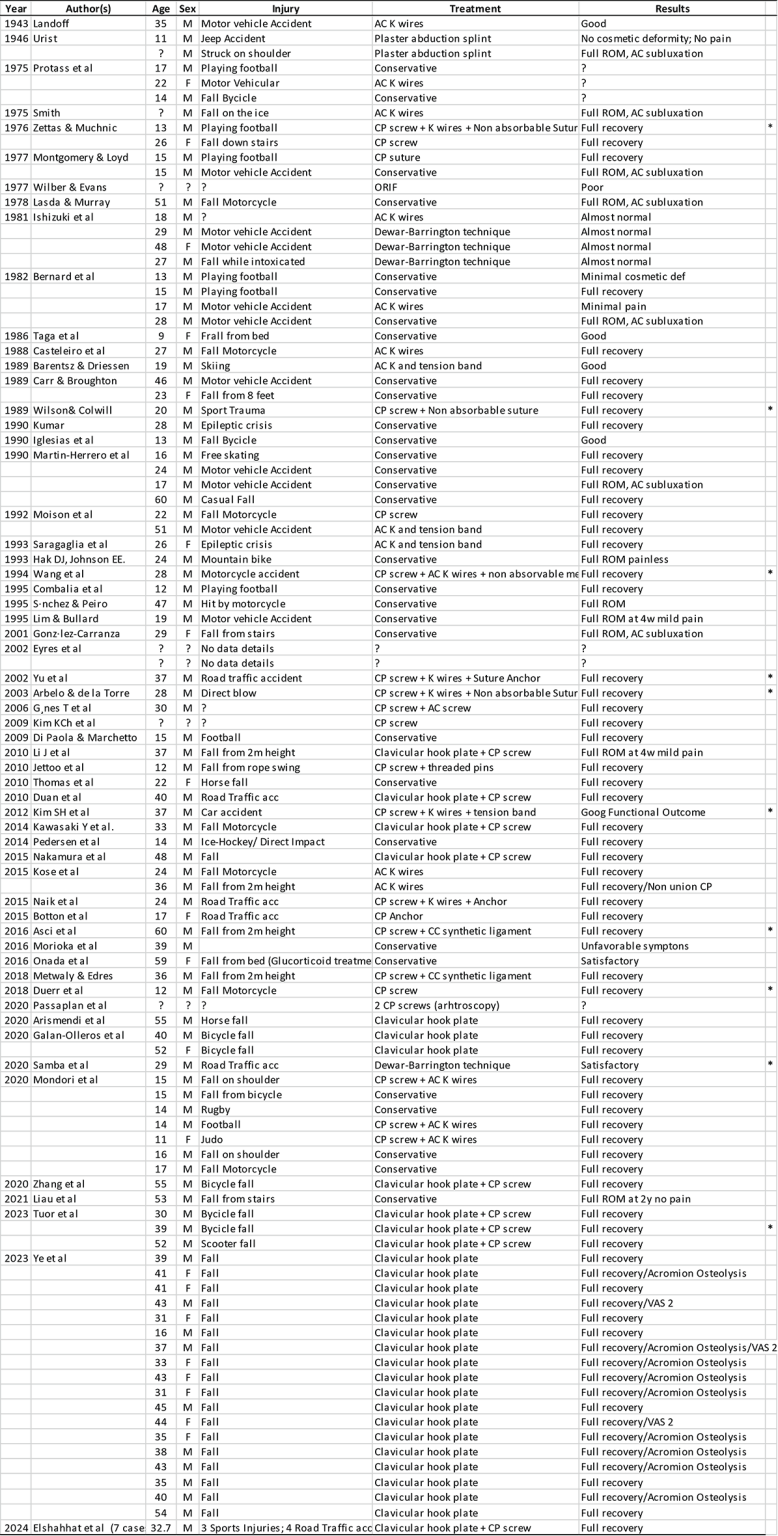
Previously published cases of Acromioclavicular (AC) dislocation associated with coracoid process (CP) fracture. [M: male; F: female; K: Kirschnner wires/Knowles wires;?: unknown data; ROM: range of motion; * reported cases with associated Coracoclavicular ligament rupture (triple injury); VAS: visual Analog Scale

**Table 3:**
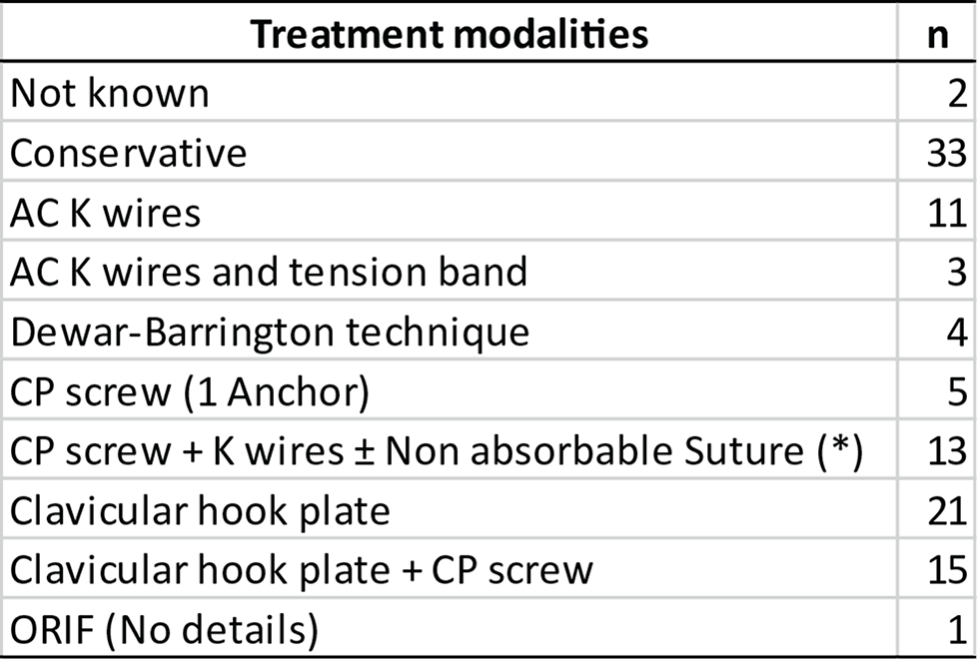
Techniques used to treat patients. (AC: acromioclavicular; CP: coracoid process; K: Kirschnner wires/Knowles wires; ORIF: open reduction internal fixation; * 10 cases with associated CC ligaments rupture)

Although there is no consensus about the ideal treatment choice for a concurrent AC dislocation and CP fracture, patients with type II undisplaced or minimally displaced CP fractures are usually treated conservatively. Surgical treatment seems to be recommended in cases of displaced fractures and combined double or triple injuries because it allows controlled anatomical reduction and stable fixation, which ultimately will reduce the occurrence of joint and bone motions, thus avoiding a nonunion. Surgical fixation is also ideal when the early recovery of range of motion and resumption of physical activities is desirable [41, 50, 55, 56, 62]. It has been proposed that AC fixation might be sufficient to restore stability to the SSSC when the CC ligaments are unharmed [32, 50]. Recently, the clavicular hook plate is recommended to fix the AC joint to provide stability and adequate support for the acromion while hypercorrecting the AC joint and avoiding most of the problems associated with the other fixation techniques [50, 55]. Trans-articular wires had been described by many authors to manage combined AC dislocation and CP fracture [5, 11, 13, 17, 22, 41]. This technique carries the risk of hardware migration, breakage and need for removal [52, 73]. A more stable fixation using hook plate for AC stability alone [50, 55] or in combination with CP fixation using cannulated screws also was described [32, 37, 40, 53, 56, 62] but again this technique had high complication rates including shoulder pain, discomfort and frequently acromion osteolysis that is only relieved after plate removal [55].

Regarding the type of treatment, we must consider that the integrity of the SSSC is the most important element in the suspension function of the upper limb, as the AC joint together with the CC ligaments is responsible for transmitting the forces and weight of the upper limb across the clavicle and to the thorax [50, 63, 64]. A good understanding of the biomechanics of the SSSC can help illustrate why a complete interruption of the AC joint associated with an injury at another point of the ring detaches the upper extremity from the axial skeleton. Although good results have been described in this type of combined injuries with either conservative or surgical treatment, the complex SSSC anatomic and biomechanical relationships lead to consider the injuries occurring at two points of the ring as potentially unstable lesions, especially when they involve a large displacement. That is the reason, and particularly in most recent publications, why so many authors tend to opt for surgical stabilization [55, 56, 62].

As per the current literature, there is no great difference in the long-term outcomes of patients managed conservatively or surgically [31, 32, 57]. However, as mentioned, a few earlier reports have commented on pain and cosmetic symptoms after conservative management [8, 11], including two of the patients in the present series. As we can appreciate, multiple approaches have been opted for by surgeons to deal with this combined injury; some have opted to address only the AC joint, and others have found that surgical reduction of the CP reduced the AC indirectly. Nevertheless, in the extremely rare combination with disrupted CC ligaments should always be repaired or reconstructed for an optimal functional outcome.

We conclude that when an AC dislocation is identified by clinical examination and X-rays, one should be aware of a possible fracture of the CP. It is possible this association to be more frequent than shown in literature because of the CP fracture can easily be missed out or mistaken with an unfussed epiphysis in routine anteroposterior radiography. A CT scan may be useful for a diagnosis and assessment of associated lesions. With the increased use of CT and MRI, coracoid process fractures are more frequently diagnosed [74].

## Conclusion and future directions

At the moment, there is no consensus on the optimum treatment strategy, as well as on the sole complete acromioclavicular joint dislocation type III, which can be treated conservatively or surgically with similar results [75]. The best treatment should be individualized considering age, associated injuries, and biomechanics. If surgeons opt for surgical treatment, there is no gold standard technique for the operative treatment of this combined injury, but operative treatment is evolving and is most used in more recent publications. There is a need for future investigations with a higher level of evidence and long-term follow-up to guide future treatment.

### Limitations of the study

The series presented, being one of the longest reported, is short and collected over the years. Otherwise, we summarized all available data on treating an AC dislocation with a concomitant fracture of the CP. Although this study has several limitations and available data are limited, we believe that it can contribute to our current understanding of this combined injury. There are no series that compare surgical treatment with conservative treatment, which given the rarity of the association makes it difficult. Future studies should include a bigger case series to collect more data on this rare injury. Furthermore, high-quality multicenter research comparing surgical and conservative treatment for these associated injuries will be necessary to optimize treatment protocols.

## Data Availability

For raw data access, contact with the corresponding author.
